# Modern health services utilization and associated factors in North East Ethiopia

**DOI:** 10.1371/journal.pone.0185381

**Published:** 2017-09-26

**Authors:** Getaw Walle Bazie, Mhiret Teshome Adimassie

**Affiliations:** 1 Department of Public Health, College of Medicine and Health Sciences, Wollo University, Dessie, Ethiopia; 2 Emergency Nutrition Project Team Leader, Care Ethiopia, Afar, Ethiopia; National Academy of Medical Sciences, NEPAL

## Abstract

**Background:**

Ethiopia is one of the developing countries with the poorest health status and the health services utilization is generally low with different patterns in different regions of the country. Therefore, the aim of this study was to assess utilization of modern health services and associated factors in Dessie, Ethiopia.

**Methods:**

A cross sectional study design was employed from January to March, 2015 in Dessie City. The total sample was 420 adults. Adults were selected by stratified random sampling. The strata were made using residence as urban and rural residents. The data was collected using pre-tested, interviewer administered questionnaire. The data was entered into Epi info^TM7^ software and exported to Statistical Package for Social Sciences (SPSS) version 20 Software for analysis. Binary logistic regression was used to evaluate independent effect of each variable on modern health service utilization by controlling the effect of others. The strength of association between dependent variable and independent variables was expressed by odds ratio with 95% confidence interval.

**Results:**

The overall modern health services utilization rate was 41.8%. Being Female sex, annual income greater than poverty line, poor perception of health status, high perceived severity of illness, two or more than two number of illnesses in the last 12 months prior to the survey and presence of chronic health problem were found to have a significant association with utilization of modern health services.

**Conclusion:**

Modern health services utilization was found to be low. Being female sex, annual income above poverty line, having poor perceived health status, having two or more than two illnesses, severe perceived severity of illness and having chronic health problem were found to have a statistically significant association with utilization. Therefore, efforts have to be made to increase utilization of modern health services through establishing systems like health extension workers and health development army.

## Introduction

Despite incredible improvements in health since 1950, there are still a number of challenges, which should have been easy to solve. One billion people lack access to health care systems and nearly one third of the world population couldn’t use health services due to different socio-economic and cultural reasons [1–4]. The health service utilization rate in Africa is low and sub-Saharan Africa in particular is very low ranging from only 0.2 annual visits to 2 visits [5].

Ethiopia is one of the sub-Saharan countries most affected by high disease burden reflected by the high rates of maternal and child mortality. Under the first 5-year rolling plan of the Health Sector Development Plan, the overall performance of the health sector had improved; however, the ability to deliver essential services in rural settings was less successful [6,7]. Though health service coverage is 86.7%, total outpatient utilization of government health facilities in Ethiopia suggest that, on average, there are about 0.25 visits per person per year. This is very far from the 3 visits of World Health Organization and Millennium Development Goals and is the lowest in sub-Saharan Africa [6].

Only 10 percent of persons reporting illness actually obtained treatment for their conditions from any health facility, government or private. In addition, utilization of health services during illness had shown great rural- urban differences with 9.5 percent for the rural and 14 percent in urban areas. Shortages and imbalances of human resources for health, geographical distance from health facilities, and socioeconomic factors aggravated by the poor health-seeking behaviors of the population were among the major obstacles to attaining wider access to health services. The average length of stay (ALOS) too long six point seven days and the average cost per patient–day equivalent (PDE) is very high (8.37 US $.) This figure is about five times lower than the sub-Saharan African average [8].

A lot have been tried to assess the health service utilization rate of individual services and to identify determinants of health care use for the individual services. But much was not done so far on the overall utilization of modern health services and factors associated with the use of health care. Therefore, the aim of this study was to assess utilization of modern health services and factors associated with it in the last 12 months in Dessie City, North East Ethiopia.

## Methods

### Study area and design

The study was conducted from January to March, 2015 in Dessie city. Dessie is a multi-ethnic city in Northeastern Ethiopia located 401Km away from Addis Ababa, the capital city of Ethiopia, in the Northeast and about 480 Km from the Amhara Regional capital city, Bahir Dar. It is more than a hundred and ten years old city with latitude and longitude of 11’8°N and 39’38°E with an elevation between 2470 and 2550 meter above sea level respectively [9].

It has an estimated area of 16,000 hectares with a density of 11,213.79 people per square kilometer. The total Population of the City is estimated to be 188,519 among this 97,661 are females and the rest are males. The majority of inhabitants belong to the two principal religions: Christianity and Islam. Recently the city is reorganized in ten sub-city administrations and six rural Kebeles. There are two public and three private hospitals, six public health centers, and more than 24 private clinics [10].

A community based cross-sectional study design was used to conduct the study. The study population was all adults above the age of 18 years who were living in Dessie Town for the last 12 months prior to the study. Study samples who were above 18 years of age and a member of that household for at least 12 months prior to the data collection period were included in the study and study samples who were severely ill during the time of data collection were excluded from the study.

### Sample size and sampling procedures

**Fig 1:**
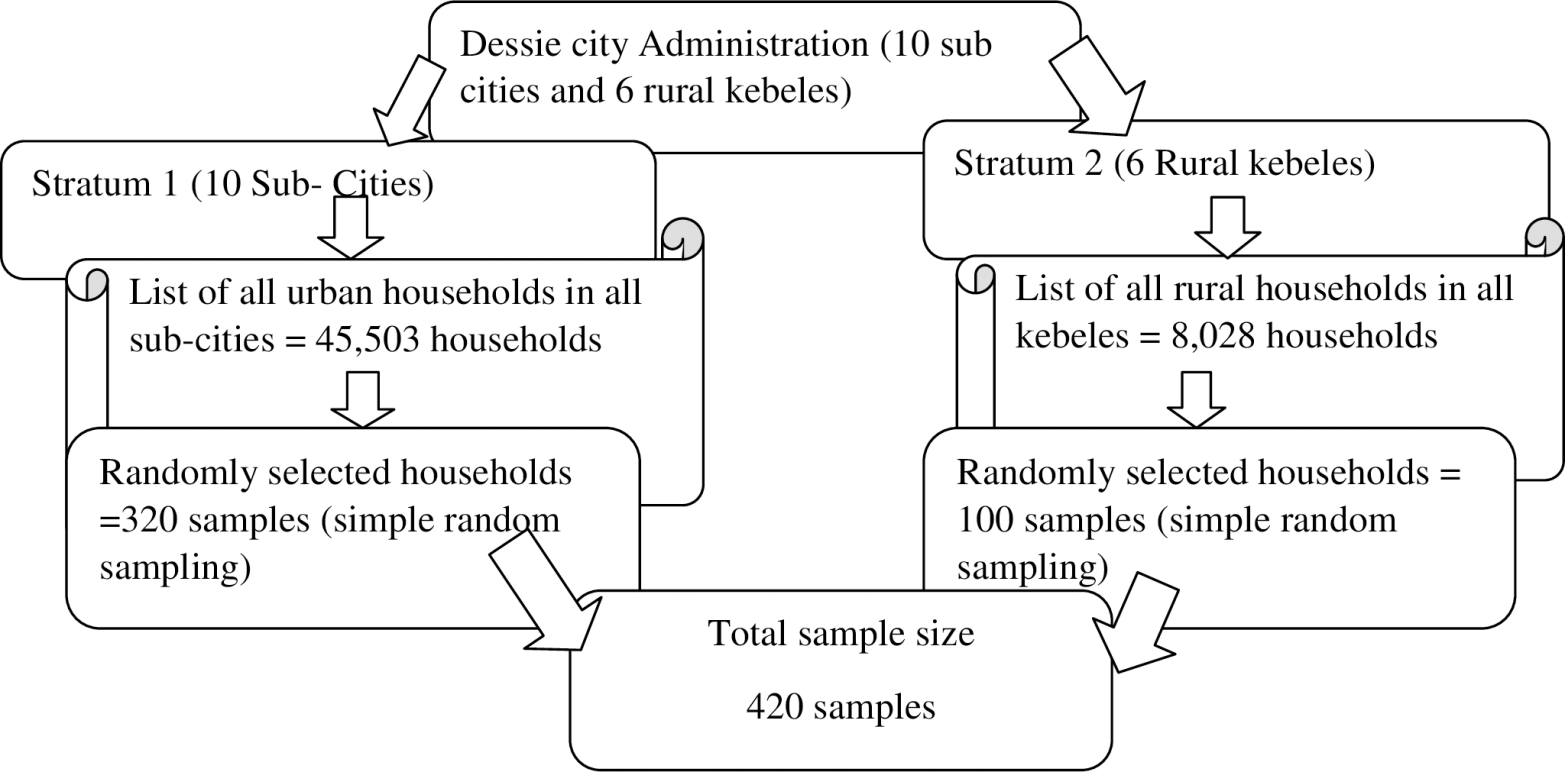
Sampling procedures.

The total sample size of 420 was calculated using single population formula and assuming, the proportion of modern health services utilization of 45.6% [11], 95% confidence level, and 5% margin of error and finally adding 10% non-response rate. Stratified random sampling was employed for selecting the study subjects. Kebeles and sub-cities were stratified into rural and urban strata. From each stratum, samples were taken proportional to their size. Households from each sub-city of urban stratum and rural stratum were selected using simple random sampling technique. The list of the households with their respective address was available in each Kebele and sub-city and the list was used as a reference frame to employ simple random sampling. The first choice for the interview was the head of the household who may be the husband or the wife who were available at the time of data collection. If both were available, the husband was interviewed. If both were not available, the member of the household above the age of 18 years and among them the older was interviewed. If none were available, next household was included. (Fig 1)

### Study variables

The dependent variable of the current study was modern health services utilization and independent variables are socio-economic variables like were age, sex, religion, educational status, income, insurance coverage, social support; health related factors like perceived health status, illness status: access related factors like travel time to the nearest health institution, perceived transport cost, perceived treatment cost, distance from the health institution.

### Operational definitions of terms

#### Modern health services

Modern health services in this study include public and private licensed health institutions (Hospitals, health centers, clinics and private non-for-profit organizations).

#### Modern health services utilization

Health services utilization in the study refers to a measure of the health of the population whether the respondent went to modern health institutions in the last 12 months prior to the study. It is a dichotomous variable based on the survey question “Did you go for health care in the last 12 months?” Yes = 1 and No = 0.

### Data collection procedures

Data was collected using pre-tested, interviewer administered questionnaire adapted from World Health Organization Manual for the Household Survey to Measure Access and Use of Medicines [12]. Twenty-Five questionnaires were pretested in Kombolcha town and corrections were made after the pretest. Study samples were interviewed by five trained data collectors. Demographic variables, socio-economic variables and variables related to access to the health services were included in the questionnaire. The questionnaire was prepared in English and was translated to the local language Amharic and translated back to English after data collection by English experts. Data collectors were given one-day training about the study and how to approach respondents, the inclusion and exclusion criteria’s, the operational definition of variables and type of variables. A field work manual was prepared before the data collection and this was used as a guide for data collection procedure.

### Data analysis

The data was entered into Epi info^TM7^ software and cleared by running each variable and exported to Statistical Package for Social Sciences (SPSS) version 20 Software for analysis. Uni-variate analysis was done to describe data in percentages, graphs, tables, means and standard deviations. Bi-variate analysis was employed to see the crude association between the independent variables and the dependent variable. Multivariate analysis using stepwise multiple logistic regression technique was done to evaluate independent effect of each variable on modern health service utilization by controlling the effect of other variables. The strength of association between dependent variable and independent variables (covariates) was expressed by odds ratio with 95% confidence interval. A P-value ≤ 0.05 was used as a cut of point for a statistically significant association.

### Ethical considerations

The study was approved by Wollo University College of Medicine and Health Sciences Ethical review committee. Support letter was obtained from the University and submitted to Dessie City Administration. Permission letter was obtained from the City Administration. Study samples were informed thoroughly about their right and the purpose of the research. Only those who gave verbal consent were interviewed. Necessary precaution was made to ensure confidentiality. The information given by the respondents was kept confidential and the names of respondents were not recorded.

## Results

The total calculated sample size was 420 and 411 of the study samples responded which gave a response rate of 97.9%. The median age of respondents was found to be 40 years. Only 11.9% of the study samples were above 65 years, 54.3% were females, 76.4% were urban residents, 97.6% were Amhara in ethnic group. Concerning religious composition, 51.1%were Muslims, 56.2% were married, 57.4% had completed secondary education and above. With regard to income, 54.3% earned below 4,383 Ethiopian Birr per annum which is below poverty line and only 7.5% did have social support (Table 1).

**Table 1:** Socio-economic characteristics of the study population, n = 411.

Variables		Frequency	Percentage
Age group	< = 24	48	11.7
	25_54	265	64.5
	55_64	49	11.9
	> = 65	49	11.9
Sex	Male	188	45.7
	Female	223	54.3
Place of residence	Rural	97	23.6
	Urban	314	76.4
Ethnicity			
	Tigrie	8	1.9
	Amhara	401	97.6
	Others^1^	2	0.2
Religion	Orthodox Christian	181	44
	Muslim	210	51.1
	Others^2^	20	4.9
Educational status	Illiterate	83	20.2
	Primary education	92	22.4
	Secondary education and above	236	57.4
Occupation	Unemployed	183	44.5
	Employed	111	27
	Owned Business organization	56	13.6
	Farmer	61	14.9
Marital status	Never Married	81	19.7
	Married	231	56.2
	Divorced/widowed	99	24.1
Husband/wife education	Illiterate	39	16.9
	Primary Education	41	17.7
	Secondary and above education	151	65.4
Partner occupation	Unemployed	77	33.3
	Employed	102	44.2
	Owned business education	35	15.2
	Farmer	17	7.4
Annual income	<187.2 US $	223	54.3
	> = 187.2 US $	188	45.7
Social support	Yes	31	7.5
	No	380	92.5
Insurance coverage	Yes	24	5.8
	No	387	94.2

Others^1^ = Oromo and Agew; Others^2^ = Protestant, Catholic, Adventist; Social support = Financial support from the community and organizations

### Utilization of modern health services

The overall modern health services utilization was found to be 41.8%. The average number of visits for health institution for the last 12 months is 1.15 with minimum of 0 and a maximum of 3 visits. (Table 2)

**Table 2:** Utilization of modern health services in Dessie Town, n = 411.

Variables		Number	Percentage
Modern health services utilization (n = 411)	No	239	58.2
	Yes	172	41.8
Type of institution (n = 172)	Government health institutions	103	59.9
	Private health institutions	66	38.4
	Other health institutions	3	1.7
Number of visits (n = 172)	Once	76	44.2
	Twice	36	20.9
	Three times	18	10.
	Four times and above	42	24.4

Regarding illness in the last 12 months prior to the study 59.1% reported that they encountered one or more acute illness. Among them, 40.7% encountered acute illness only once whereas the rest had encountered two or more times. The major manifestations of study population who had been ill in the last 12 months prior to the study were fever, diarrhea, cough, and headache. (Table 3).

**Table 3:** Types of illness in Dessie Town, n = 243.

Type of illness	Frequency	Percentage
Fever	39	16.05
Diarrhea	37	15.22
Cough	97	39.92
Headache	59	24.28
Others	11	4.53
Total	243	100.0

The most common reason for not visiting health institutions was the perception that the illness was not much severe. Next is economic reason and more than half of the respondents had more than one reason for not visiting modern health institutions (Table 4).

**Table 4:** Common reasons for not visiting the health institutions in the last 12 months (n = 239).

	Frequency	Percentage
Illness couldn't be treated at the institution	22	9.2
Illness is not severe enough	69	28.9
Have no money to pay for health care	65	27.2
Perceived quality of health institutions is poor	47	19.7
The institution is far	3	1.3
Long service time	13	5.4
Lack of laboratory facilities	2	.8
Had taken home treatment	11	4.6
Bought drugs from drug vendors	1	.4
Visited traditional healers	2	.8
No reason	3	1.3
Others^3^	1	.4
Total	239	100.0

Others^3^ = don’t like to go to health institution

### Factors associated with modern health services utilization

After controlling for confounders on multivariate logistic regression, being female sex, annual income above poverty line, having poor perceived health status, having two or more than two illnesses, severe perceived severity of illness and having chronic health problem were found to have a statistically significant association with utilization.

Females were 4.071 times more likely to utilize modern health services than males (95% CI 1.074, 15.430). Adults above the poverty line were 4.026 times more likely to use the health services as compared to those below the poverty line (95% CI 1.243, 13.033). Adults who perceived that their health status was poor were 76.923 times more likely to visit the health institution than those who perceived their health status was good (95% CI 20.408, 333.333).

Similarly, among those who were ill in the last 12 months prior to the study those who perceived that their illness was severe were 21.486 times more likely to visit than those perceived mild illness (95% CI 5.88, 78.499). Adults who encountered acute illness twice and above in the last 12 months were 5.780 times more likely to use health services than who encountered only once (95% CI 1.639, 20.408). Adults with chronic health problem were 4.247 times more likely to use modern health services than those without chronic health problem (95% CI 1.176, 15.335). (Table 5)

**Table 5:** Factors associated with modern health service utilization in Dessie Town.

Variables		Modern Health Services Utilization				
		Yes	No	Total	COR (95% CI)	AOR (95% CI)
**Sex**	Female	105	118	188	1.607 (1.08,2.392)*	4.071 (1.074,15.430)**
	Male	67	121	223	1	
**Annual income**	> = 4383 Birr	120	68	223	5.803(3.775,8.920)*	4.026 (1.234,13.033)**
	< 4383 Birr	52	171	188	1	
**Perceived Health status**	Poor	153	13	166	139.992 (66.667,333.333)*	76.923 (20.408, 333.333)**
	Good	19	226	245	1	
**Perceived Severity of illness**	Severe	119	21	140	30.8 (15.2,62.5)*	21.486 (5.881,78.499)**
	Mild	16	87	103	1	
**Number of Illnesses**	Once	55	44	99	1	
	Twice and above	80	64	144	1(0.607,1.672)	5.780 (1.639, 20.408)**
**Chronic health problem**	Yes	114	39	153	10.08 (6.322,16.071)*	4.247 (1.176, 15.335)**
	No	58	200	258	1	

COR = Crude odds ratio; AOR = Adjusted odds ratio; 1 = Reference category

* Significant association (crude)

**Significant association (Adjusted)

## Discussion

In this study, modern health services utilization rate in the previous 12 months was found to be 41.8% and the average number of visits to the health institutions were found to be 1.15 times. The average number of visits was less than by half and more from the world health standard which is 3 times per annum [6]. The magnitude of health service utilization was different from previous findings.

This finding is larger when compared to the finding in Nigeria (7.6%) [13]. This might be due the study in Nigeria focus on primary health care in low-income settings whereas our study focused on utilization of modern health services in all settings and beyond primary health care services. In a neighbor country Kenya this was 53% [14] which is greater than the current finding. The reason for this difference might be due to recall bias as the current study assessed the last 12 months of utilization but the study in Kenya had only 3 months of recall period.

This finding was also higher than the national utilization rate of 10% [8]. The possible justification is the current study focused urban dwellers and rural dwellers which are near to the town and that there is almost a decade of time difference. This finding was also a little higher than reported in Amhara region and almost similar with a study in Jimma Zone, 38.7% and 45.6% respectively [11, 15]. This is because the current study focused on the overall utilization while the study on Amhara region did only on terminally ill patients and there is large time difference between the two studies as nowadays the health infrastructure dramatically changed nationally.

In this study, sex, annual income, perceived health status, number of illnesses, perceived severity of illness and chronic health problem showed significant association with modern health services utilization.

Females were found to utilize the health services 4.071 times more likely than males. This might be explained by the fact that women are more prone to illness due to peculiar reproductive health needs. Moreover, women would be more likely to accompany their children to health institutions where they seek treatment for themselves too. This finding is consistent with the study done by Fistum and Challi et al [11].

Annual income was also found to have significant association with utilization of modern health services. Participants above the poverty line were 4.026 times more likely to use the health services as compared to those below the poverty line. It was as expected in that those who could afford the cost of the service and related expenditures are more likely to utilize as this was confirmed by study in Southern Brazil in that income was a determinant factor with a 10% reduction in visiting a doctor for the poor as compared to intermediate and above income. This was also consistent in studies in Addis Ababa and Jimma Zone [11, 16, 17].

Perceived health status was also found to be associated with utilization of the health services. Respondents who perceived that their health status was poor were more likely to visit the health institution than those who perceived that their health status was good. This might also be as expected in that those who perceived that their health was in poor condition seeks treatment and would visit the health institution.

The other important variable which had shown statistically significant association was number of illness in the last 12 months. Those individuals who encountered illness twice and above were more likely to use health services than those who encountered illness only once. The possible reason might be people do not go to health institutions unless they become ill repeatedly. The association of perceived severity of illness was also expected. Because those who were severely ill are ordered to go to the health institution for relief unless uncontrollable issues are challenged.

Chronic health problem had also shown statistically significant association. Those who had chronic health problem were more likely to use health services than those who did not have chronic health problem. This is plausible in that, people with a known chronic health problem would have follow ups at the health institution and those who had the problem are more often to visit the health institution. This in line with a study conducted in Jimma Zone [11].

The questionnaire was administered by an interviewer; there might be a possibility of social desirability bias. For example, the modern health service utilization rate might be overestimated, as many people might be afraid to admit that they visited a traditional practitioner. The second limitation worth mentioning was recall bias since evaluation of self-reported behavior patterns was retrospective. Twelve months’ period of recall might be long as compared to some of the studies. So that utilization of health services and number of illness episodes might be underestimated due to recall bias.

## Conclusion

Modern health services utilization was found to be low. Being female sex, annual income above poverty line, having poor perceived health status, having two or more than two illnesses, severe perceived severity of illness and having chronic health problem were found to have a statistically significant association with utilization.

## Recommendations

Efforts have to be made to increase utilization of modern health services through establishing systems like health extension workers and health development army. Our country, Ethiopia should develop policies that will change C the perception of people towards seeking health services has to be made.

## Supporting information

S1 File Questionnaire. (DOC)
