# Supplementary material for: Modern health services utilization and associated factors in North East Ethiopia
Source: PLoS One. 2017 Sep 26;12(9):e0185381. doi: 10.1371/journal.pone.0185381 (PMC5614575; doi:10.1371/journal.pone.0185381)
Supplement: S1 File — (DOC) [file pone.0185381.s001.doc]

**English Version Questionnaire**

**Consent form**

Hello my name is _________________________________I am collecting information on the Magnitude of health service utilization and factors influencing it. The true answer that you give for us means a lot to achieve the goal of the research. We want to ask you about different things relating to you and your families about health service utilization. Whatever information you provide will be kept strictly confidential. No information identifying you will ever be realized to anyone outside of this information collection activity. Participation in this survey is voluntary and you can choose not to answer any individual question or all of the questions. You may also stop the interview completely at any time without any consequences at all. However, we encourage you to participate in this study since the results will help a lot for different public health interventions so far.

At this time, do you want to ask me anything about the purpose or content of this interview?

May I begin the interview now?

Signature of interviewer: ___________________________

Date of data collection**________________** Code number ___________

Data collector name _____________________

**Questionnaire**

**Circle questions that have choices and write on space provided for others.**

| **Part-1 Socio-demographic and economic condition of the respondent** | | | | | | | **Remark(**write any comment in front of each question) |
| --- | --- | --- | --- | --- | --- | --- | --- |
| 1 | | Age | ________________(year)  99. I don’t know | | | |  |
| 2 | | Sex | 1. Female  2. Male | | | |  |
| 3 | | Place of residence | 1. Rural 2. Urban | | | |  |
| 4 | | Ethnicity | 1. Oromo  2. Tigre  3. Amhara  4. Afar  Others_____________________ | | | |  |
| 5 | | Religion | 1. Orthodox  2. Muslim  4. Catholic  5. Others_____________________ | | | |  |
| 6 | | What is your educational status? | 1. Can’t read and write  2. Can Read and write  3. Complete 1-4 grade  4. Completed 5-8 grade  5. Completed 9-12 grade  6. completed above grade 12  7. other---------------------------------- | | | |  |
| 7 | | What is your occupation? | 1. Un employed* 2. Student 3. Day worker 4. Government employee 5. Private employee 6. Merchant or have own business organization 7. If others specify…………………………. | | | | ***=** not involved in any money earning mechanism |
| 8 | | What is your Marital status? | 1. Single  2. Married  3. Living together  4. Divorced  5. Separated  6 Widowed/widower | | | |  |
| 9 | | What is your spouse occupation? | 1. Unemployed* 2. Student 3. Day worker 4. Government employee 5. Private employee 6. Merchant or has own business organization 7. If others specify…………………………. | | | | ***=** not involved in any money earning mechanism |
| 10 | | How many people live in your house hold including you? | _______________________ | | | |  |
| 11 | | How much is your family average monthly income/economic status? | ____________________________ | | | |  |
| 12 | | Do you have any social support gained in kind or cash in the family for the in the last 12 months? | 1. Yes 2. No | | | |  |
| 13 | | If your answer for Q 12 is ‘yes’ how do you rate the support? | 1. Extensive 2. Enough 3. Minimal | | | |  |
| 14 | | Do you have a health insurance coverage | 1. Yes 2. No | | | |  |
| **Part 2 The health status of the individual** | | | | | | | |
| 15 | | How do you rate your health status? | 1. Very poor 2. Poor 3. Fair 4. Good 5. Very good | | | |  |
| 16 | | Have you encounter any acute illness in the last 12 months | 1. Yes 2. No | | | | *Acute illness is a disease with an abrupt onset, and usually a short course* |
| 17 | | If your answer for Q 16 is ‘Yes’ what was the major manifestations? | 1 Fever  2 Diarrhea  3 Acute respiratory infection  4 Headache  5 If others specify………………………………. | | | |  |
| 18 | | If your answer for Q 16 is ‘Yes’ how many times did you encounter it? | 1 once  2 twice  3 three times  4 four times and above | | | |  |
| 19 | | If your answer for Q 16 is ‘Yes’ how severe was it? | | 1. Severe 2. Moderate 3. Mild | | |  |
| 20 | | Do you have any chronic illness? | 1. Yes 2. No | | | | *Chronic illness is a long-lasting condition that can be controlled but not cured. E.g. Diabetics, any type of cancer, hyper tension* |
| **Part 3 Questions related to access to health institutions** | | | | | | | |
| 21 | | How far is the health institution from here (in km)? | | | _______________________ | |  |
| 22 | | How long does it take in hours to reach there? | | | ________________________ | |  |
| **Part 4 Questions related to health care utilization** | | | | | | | |
| 23 | Did you visit modern health institutions for the last 12 months? | | | | | 0 Yes  1 No |  |
| 24 | If your answer for Q 23 is ‘Yes’ which specific health institution did get visited? | | 1. Health center 2. Government Hospital 3. Health post 4. Private Hospital 5. Private clinic 6. Private-non-for profit organizations | | | | *Modern health institution include private and government hospitals, health centers, health posts , private clinics and private non-for- profit organizations* |
| 25 | If your answer for Q23 is ‘yes’ how many times did you visit the health institution? | | 1 once  2 twice  3 three times  4 four times and above | | | |  |
| 26 | If your answer for 23 is ‘No’ where did you get treated/visit regularly? | | 1. Not treated at all 2. Visited a drug vendor 3. Home treated 4. Religious institutions 5. Traditional healing institutions 6. If others specify……………………………. | | | |  |
| 27 | What is your perception on the treatment cost of in the modern health institutions that you visited? | | 1 expensive  2 Medium  3 Cheap | | | |  |
| 28 | What is your perception on the transport cost to reach the modern health institution? | | 1. expensive 2. Medium 3. Cheap | | | |  |
| 29 | If your answer for Q 23 is ‘NO’ what is your main reason that you didn’t visit modern health institutions? | | Because:   1. My illness couldn’t be treated at the institution 2. My illness is not severe enough 3. I didn’t have money to pay for health care 4. The perceived quality of health institutions is poor 5. The institution is far from me 6. Long service time 7. Lack of laboratory facilities 8. I had taken home treatment 9. I bought drugs from drug vendors 10. I visited traditional healers 11. I visited religious institutions 12. No reason 13. Other reason…………………………… | | | |  |

Thank you for your attention and if you have concerns about this interview and the study you can specify here……………………………………………………………………………………………………………………………………………………………………………………………….

**Thank You for Your Attention**

**Questionnaire (Amharic Version)**

**በደሴ ከተማ አስተዳደር የነዋሪዎችን በህመም ወቅት ያለውን የጤና ተቋማት አጠቃቀም ሁኔና ምክንያቶችን ለማጥናት የተዘጋጀ መጠይቅ**

**መግቢያ፡**

እኔ ………………………..እባለለሁ፡፡ የነዋሪዎችን በህመም ወቅት ያለውን የጤና አጠቃቀም ሁኔና ምክንያቶችን ለማት ይረዳ ዘንድ መረጃ በመሰብሰብ ላይ እገኛለሁ፡፡ እርስዎ የሚሰጡኝ ትክክለኛ መረጃ ለዚህ ጥናት መሳካት ወሳኝ ነው፡፡ በተጨማሪም ከጥናቱ በሚገኙት ውጤቶች አማካኝነት በሚወሰኑ ውሳኔወችና ተግባራት እርስዎ፣ ቤተሰብዎና የአካባቢው ህብረተሰብ ተጠቃሚ ይሆናሉ፡፡ ማንኛውም የሚሰጡን መረጃ በሚስጥር ይያዛል፡፡ ስምዎትን መጻፍም ሆነ መናገር አይጠበቅብዎትም፡፡ በዚህ ጥናት የሚሳተፉት ፈቃደኛ ከሆኑ ብቻ ነው፡፡ ፈቃዳ ሆነው መረጃ መስጠጥ ጀምረው ያልተመቸ ነገር ቢያጋጥምዎት መጠየቅ ወይም ጥናቱ ማቋረጥ ይችላሉ፡፡ አሁን ስለሰጠሁዎት መረጃና ስለ ጥናቱ የሚጠይቁኝ ነገር ካለ መጠየቅ ይችላሉ፡፡

አሁን መጠይቁን መጀመር እችላለሁ?

ቃለመጠይቁን ያስሞላው/ የሞላው ስም………………………………………………………..

መረጃው የተሰበሰበበት ቀን……………………………….መለያ ቁጥር…………………………

**መመሪያ፡** ከጥያቄው ፊት ለፊት ከተቀመጡት አማራጮች የመረጡትን ያክብቡ ወይም በተቀመጠው ክፍት ቦታ ላይ የእርስዎን መልስ ያስቀምጡ፡፡

| **ክፍል አንድ፡ ማህበራዊ፣ ዲሞግራፊያዊና የኢኮኖሚ ሁኔታን የተመለከቱ ጥያቄዎች** | | | | | | | | | | | | | **ምርመራ (ማንኛውንም አስተያየት በዚህ ሳጥን ላይ ይግለጹ)** | | | |
| --- | --- | --- | --- | --- | --- | --- | --- | --- | --- | --- | --- | --- | --- | --- | --- | --- |
| 1 | | ዕድሜ | ________________(በዓመት)  99. አላውቀውም | | | | | | | | | |  | | | |
| 2 | | ጾታ | 1. ሴት  2. ወንድ | | | | | | | | | |  | | | |
| 3 | | የመኖሪያ ቦታ | 1. ገጠር 2. ከተማ | | | | | | | | | |  | | | |
| 4 | | ብሄር | 1. ኦሮሞ  2. ትግሬ  3. አማራ  4. አፋር  5. ሌላ ቢሔር ይጠቀስ________________ | | | | | | | | | |  | | | |
| 5 | | ሐይማኖት | 1. ኦርቶዶክስ  2. ሙስሊም  3. ፕሮቴሰታንት  4. ካቶሊክ  5. ሌላ ሃይማኖት ይጠቀስ_____________ | | | | | | | | | |  | | | |
| 6 | | የትምህርት ደረጃ | 1. ማንበብና መጻፍ የማይችል/ትችል 2. ማንበብና መጻፍ የሚችል/ትችል   3 ከ1ኛ እስከ 4ኛ ክፍል የተማረ/ች  4 ከ5ኛ እስከ 8ኛ ክፍል የተማረ/ች  5. የሁለተኛ ደረጃ ትምህርት (ከ 9 እስከ12 ያተናቀቀ/ች  6. ከ 12 ክፍል በላይ የተማረ/ች  7. ሌላ ካለ ይጠቀስ………………………. | | | | | | | | | |  | | | |
| 7 | | ስራህ/ሽ ምንድን ነው? | 1. ምንም ዓይነት ስራ የሌለው *(ለምሳሌ የቤት እመቤት*) 2. ተማሪ 3. የቀን ሰራተኛ 4. የመንግስት ሰራተኛ 5. የግል ድርጅት ሰራተኛ 6. ነጋዴ ወይም የድርጅት ባለቤት 7. ሌላ ካለ ይጠቀስ………………………… | | | | | | | | | |  | | | |
| 8 | | የጋብቻ ሁኔታ? | 1. ያላገባ/ች  2. ያገባ/ች  3. በጓደኝነት ያለ/ች  4. አግብቶ የፈታ/ች  5. ተለያይተው የሚኖሩ  6 ባል/ሚስት የሞተባት/የሞተበት | | | | | | | | | |  | | | |
| 9 | | የጓደኛ/ የስራ ሁኔታ ? | 1. ምንም ዓነት ስራ የሌለው *(ለምሳሌ የቤት እመቤት*) 2. ተማሪ 3. የቀን ሰራተኛ 4. የመንግስት ሰራተኛ 5. የግል ድርጅት ሰራተኛ 6. ነጋዴ ወይም የድርጅት ባለቤት 7. ሌላ ካለ ይጠቀስ……………………… | | | | | | | | | |  | | | |
| 10 | | እርስዎን ጨምሮ የቤተሰቡ አባላት ምን ያህል ናችሁ? | ______________________ | | | | | | | | | |  | | | |
| 11 | | በአማካኝ በወር ምን ያህል ገቢ ያገኛሉ? | ___________________ | | | | | | | | | |  | | | |
| 12 | | ከቤተሰቡ ውስጥ በአይነት ወይም በጥሬ በገንዘብ የማህበራዊ ድጋፍ የሚያገኝ አለ? | 1. አዎ 2. የለም | | | | | | | | | |  | | | |
| 13 | | በተራ ቁጥር 12 ለተጠየቀው ጥያቄ መልስዎ “አወ” ከሆነ ድጋፉን እንዴት ያዩታል? | | | 1. ከበቂ በላይ 2. በቂ 3. አነስተኛ | | | | | | | |  | | | |
| 14 | | የጤና መድህን ሽፋን አለዎት? | | | | | 1. አዎ 2. የለም | | | | | |  | | | |
| **ክፍል ሁለት፡ የግል የጤና ሁኔታን በተመለከተ** | | | | | | | | | | | | | | | | |
| 15 | የጤና ሁኔታወን እንዴት ያዩታል? | | | | | | | | 1. ጥሩ አይደለም 2. ጥሩ ነው |  | | | | | | |
| 16 | ባለፉት 12 ወራት ውስጥ ማንኛውም አይነት ድንገተኛ በሽታ ወይም ህመም አሞዎት ያውቃል | | | | | 1. አወ 2. የለም | | | | *ድንገተኛ በሽታ ማለት በአጋጣሚ ወይም በታወቅ ምክንያት የሚያምና የረጅም ጊዜ ክትትል የማያስፈልገው ታክሞ ሊድን የሚችል ማለት ነው፡፡* | | | | | | |
| 17 | በተ/ቁ 16 ለተጠየቁት ጥያቄ መልስዎ “አዎ” ከሆነ ዋና ምልክቶቹ ምን ነበር | | | | | 1. ትኩሳት 2. ተቅማጥ 3. ድንገተኛ የመተንፈሻ አካል በሽታ 4. ራስ ምታት 5. ሌላ ካለ ይጠቀስ………………… | | | | | |  | | | | |
| 18 | በተ/ቁ 16 ለተጠየቁት ጥያቄ መልስዎ “አዎ” ከሆነ ተመሳሳ ወይም ሌላ ህመም ምን ያህል ጊዜ ያውቅወታል? | | | | | 1. አንድ ጊዜ ብቻ 2. ሁለት ጊዜ 3. ሶስት ጊዜ 4. አራት ጊዜና ከዚያ በላይ | | | |  | | | | | | |
| 19 | በተ/ቁ 16 ለተጠየቁት ጥያቄ መልስዎ አዎ የህመሙን ስሜት እንዴት ይገልጹታል? | | | | | 1. ስቃይ አለው 2. መጠነኛ ስቃይ አለው 3. ስቃይ የለውም | | | |  | | | | | | |
| 20 | የቆየ በሽታ አለበዎት? | | | | | 1. አዎ 2. የለም | | | | *የቆየ በሽታ ማለት በሃኪም የተነገረዎት ሊጠነቀቁለት ነገር ግን ሊድን የማይችል ማለት ነው፡፡ለምሳሌ የስኳር በሽታ፣ ማንኛውም ዓይነት ካንሰር፣የደም ግፊት* | | | | | | |
| **ክፍል ሶስት፡ የጤና ተቋማት ሁኔታ በተመለከተ** | | | | | | | | | | | | | | | | |
| 21 | | የጤና ተቋሙ ርቀት ከዚህ በኪሎ ሜትር በግምት ስንት ይሆናል? | | | | _______________ | | | | | | | |  | | |
| 22 | | ከዚያ ለመድረስ በሰዓት በግምት ስንት ይሆናል? | | | | ………………… | | | | | | | |  | | |
| **ክፍል አራት የጤና ተቋማት አጠቃቀም ሁኔታን በተመለከተ** | | | | | | | | | | | | | | | | |
| 23 | | ባለፉት 12 ወራት በዘመናዊ ጤና ተቋማት ለራስዎ ጉዳይ ሂደው ያውቃሉ? | | | | | | 0 አዎ  1 የለም | | | *ዘመናዊ የጤና ተቋም ሲባል የግል ሆስፒታሎችና ክሊኒኮች፣ የመንግስት ጤና ጣቢያዎችን፣ ሆስፒታሎችንና ጤና ኬላዎችን እንዲሁም ለትርፍ ያልተቋቋሙ የግል ድርጅቶችን ያጠቃልላል* | | | | | |
| 24 | | በተ/ቁ 24 ለተጠየቀው ጥያቄ መልስዎ “አዎ” ከሆነ የትኛው የጤና ተቋም? | 1. ጤናጣቢያ 2. የመንግስትሆስፒታል 3. ጤናኬላ 4. የግልሆስፒታል 5. የግልክሊኒክ 6. ለትርፍ ያልተቋቋመ የግል ድርጅት 7. ሌላካለይጠቀስ…………………… | | | | | | | |  | | | | | |
| 25 | | በተ/ቁ 24ለተጠየቀውጥያቄመልስዎ “አዎ”ከሆነባለፉት 12 ወራትስንትጊዜሂደዋል? | | | | | 1. አንድ ጊዜ ብቻ 2. ሁለት ጊዜ 3. ሶስት ጊዜ 4. አራት ጊዜና ከዚያ በላይ | | | | *ከህክምናየተመላለሱባቸውንጊዜያትይግለጹ* | | | | | |
| 26 | | በተ/ቁ 24ለተጠየቀውጥያቄመልስዎ “የለም”ከሆነ ብዙ ጊዜ (አዘውትረው) የሚሔዱት የትነው? | | 1. አልታከምሁም 2. ከመድሃኒትማደያመድሃኒትበመግዛት 3. የመኖሪያቤትውስጥህክምና 4. ወደሃይማኖትተቋምበመሄድ 5. ከባህላዊመድሃኒት አዋቂዎችበመሄድ 6. ከላይከተዘረዘሩትውጬ ከሆነ ይጠቀስ…………………………………. | | | | | | | | | | |  | |
| 27 | | ዋጋቸው (አጠቃላይ ወጭውን ሲገመግሙትምንይመስላል? | | | | | 1 ውድነው  2 መካከለኛ  3 ርካሽነው | | | |  | | | | | |
| 28 | | ወደ ጤናተቋምለመድረስየሚፈጀውንየትራነስፖርትዋጋስእንዴትያዩታል? | | | | | 1. ውድነው   2 መካከለኛ  3 ርካሽነው | | | |  | | | | | |
| 29 | | በተራቁጥር 25 ለተጠየቀውጥያቄመለስዎ“የለም” ከሆነወደ ጤና ተቋማትሂደውየማይገለገሉበትትልቁምክንያትምንድንነው? | | | | | **ምክንያቱም፡**   1. ያመመኝ ህመም በዘመናዊ ህክምና ተቋም ታክሞ ሊድን የሚችል አይደለም 2. ያመመኝህመምአስጊስላላይደለ 3. ገንዘብስላልነበረኝ 4. የጤናተቋማቱአገልግትአሰጣጥጥራትአርኪስላልሆነ 5. የጤናተቋሙስለሚርቀኝ 6. በጤና ተቋሙ ተራ ቶሎ ስለማይደርሰኝ 7. የላቦራቶሪ ምርመራ ስለሌለ 8. ቤቴስለምታከም 9. መድሃኒት ከመድሃኒት መደብር ገዝቼ ስለምጠቀም 10. ወደ ባህላዊ መድሃኒት አዋቂ ስለምሄድ 11. ወደ ሃይማኖት ተቋት ስለምሄድ 12. ምንም ምክንያት የለኝም 13. ሌላምክንያትካለይጠቀስ……………. | | | | | | | | |  |

በጥናቱ ወይም በመጠይቁ ላይ ሌላ ተጨማሪ አስተያየት ወይም ሃሳብ ካለዎት ይግለጹ ………………………………………………………………………………………………………………………………………………………………………… **ስለትዕግስትዎ ከልብ እናመሰግናለን፡፡**
